# Human umbilical cord mesenchymal stem cells implantation accelerates cutaneous wound healing in diabetic rats via the Wnt signaling pathway

**DOI:** 10.1186/s40001-019-0366-9

**Published:** 2019-02-08

**Authors:** Yanfu Han, Tianjun Sun, Yanqing Han, Lingling Lin, Chang Liu, Jing Liu, Guangzhi Yan, Ran Tao

**Affiliations:** 1 Department of Plastic Surgery, Beijing Shijitan Hospital, Capital Medical University, 10 Tieyilu, Yangfangdian Haidian District, 100038 Beijing, People’s Republic of China; 2 Department of Burn and Plastic Surgery, Hainan Branch of People’s Liberation Army General Hospital, Haitangwan, Sanya, People’s Republic of China; 3 School of Electrical and Information Engineering, Wuhan Institute of Technology, 366 Huquan, Wuhan, People’s Republic of China; 4 Department of Burn and Plastic Surgery, Harrison International Peace Hospital, 180 Renmin East Road, Hengshui, Hebei People’s Republic of China; 5 Department of Plastic Surgery, PLA General Hospital, 28 Fuxing Road, Beijing, People’s Republic of China

**Keywords:** Mesenchymal stem cell therapy, Tissue engineering skin, Diabetes mellitus, Wound healing, Wnt signaling pathway

## Abstract

**Objective:**

Difficulty in wound healing is one common complication of diabetes mellitus. The study explored whether the therapeutic effect of human umbilical cord mesenchymal stem cells (hUCMSCs) on diabetic ulcer wound was enhanced by the activation of the Wnt signaling pathway.

**Methods:**

Rat diabetic model was established by intraperitoneal injection of Streptozotocin (STZ). hUCMSCs were purified and seeded on the collagen–chitosan laser drilling acellular dermal matrix (CCLDADM) scaffold, which was subsequently implanted into the cutaneous wound of normal and diabetic rats, followed by daily injection of Wnt signaling pathway agonist (Wnt3a) or antagonist (sFRP3) at the edge of the scaffold. Wound healing was checked on days 7, 14, and 21, and the fibrous tissue deposition, capillaries, and epidermal regeneration at the wound were examined by hematoxylin–eosin staining. The hUCMSCs-CCLDADM scaffold was cultured in vitro and treated with Wnt3a or sFRP3, followed by evaluation of cell proliferation, cell proliferation rate, survival status, and altered protein levels in the Wnt signaling pathway using BrdU staining, CCK-8 assay, live/dead staining, and Western blotting, respectively.

**Results:**

On days 7 and 14 postoperatively, the speed of wound healing was significantly lower in diabetic rats than that in normal control rats. This phenomenon was significantly improved by the activation of the Wnt signaling pathway that also elevated the fibrous protein deposition and the abundance of capillary in the granulation tissue. Conversely, blockade of Wnt signaling slowed the healing of skin wound in diabetic rats. The activation of Wnt signaling pathway promoted the proliferation and differentiation and decreased the apoptosis of hUCMSCs, thereby elevating the number of living hUCMSCs on the CCLDADM scaffold, while the suppression exerted a contrary effect.

**Conclusion:**

The activation of the Wnt signaling pathway promotes the healing of diabetic skin wound by the regulation of proliferation and differentiation of hUCMSCs on the CCLDADM scaffold.

## Background

Diabetic ulcer is one of the major complications of diabetes that accounts for about 36% of all chronic skin ulcers, and the amputation rate is up to 19.03% in individuals with diabetic ulcers [1]. A chronic diabetic ulcer is characterized by complex etiology, prolonged treatment duration, high medical expense, predisposition to repeated recurrence, and poor clinical prognosis. Therefore, to improve the quality of patient’s life, identifying the effective therapeutic measure for diabetic chronic skin ulcer wound is essential [2].

Mesenchymal stem cells (MSCs) are adult stem cells with the potential of multilineage differentiation; MSCs are primarily derived from the bone marrow, fat, and umbilical blood and cord [3, 4]. Human umbilical cord mesenchymal stem cells (hUCMSCs) possess characteristics of robust proliferation and differentiation, as well as weak immunogenicity, thereby posing a great potential in the field of tissue engineering [5]. To improve the survival ratio of hUCMSCs on the wound surface, we implanted hUCMSCs on the scaffold made of the collagen–chitosan laser drilling acellular dermal matrix (CCLDADM), which was then spread on the wound surface, to achieve the optimal therapeutic effect on the wound repair [6]. Therefore, whether this method could be feasible for the management of refractory diabetic ulcer wound necessitates further exploration.

Wnt signaling pathway is critical for the regulation of cell proliferation and differentiation, as well as posttraumatic tissue regeneration and repair [7]. A recent study reported that Wnt signal was attenuated in the skin wound of diabetic rat model, and its suppression slowed down the healing speed, thereby implying Wnt signaling pathway as a vital element for the regulation of diabetic skin ulcer [8]. Therefore, the current study deployed the hUCMSCs-loaded scaffold for treatment of diabetic skin ulcer wound and explored the activation of Wnt signaling pathway in hUCMSCs seeding on the scaffold to accelerate wound healing.

## Reagents and methods

### Experimental animals and main reagents

Three-month clean-class male Sprague–Dawley (S–D) rats were purchased from Beijing Vital River Laboratory Animal Technology Co. (Beijing, China). Purified Wnt3a and secreted frizzled-related proteins (sFRP3) proteins were purchased from Abnova. hUCMSCs, culture media (HUXUC-90011), BrdU cell proliferation assay kit (KGA319-02), and anti-Cyclin-D1 (KG22272-1), anti-c-myc (KG21035-1), anti-p63 (KG22608), and anti-PCNA (KG22625) antibodies were purchased from Cyagen Biosciences (China). Insulin (I0908), retinoic acid (R2625), and CaCl_2_ (793639) were purchased from Sigma. Anti-CK19 antibody (AF0192) was obtained from Affinity, while anti-GAPDH antibody was purchased from CST. CCLDADM scaffold was house-accustomed during previous studies. Streptozotocin (STZ) was purchased from Aladdin (China) (S110910), FBS (10438034) from GIBCO, DAPI (E607303) from Sangon Biotech Co., Ltd. (Shanghai, China), and epidermal growth factor (AF-100-15) and basic fibroblast growth factor (100-18B) were purchased from Peprotech.

### Methods

#### Establishment of the cutaneous wound model in diabetic rats

Rat diabetic model was established by intraperitoneal injection of STZ (65 mg/kg). The successful establishment of the diabetic model was determined by three consecutive measurements of glycemia ≥ 16.7 mmol/L, using normal SD rats as the control. Under aseptic conditions and anesthesia via intraperitoneal injection of 2.5% pentobarbital sodium, the whole layers of dorsal skin were resected in the control group (group A, 9 rats) and diabetic group (group B/C/D, 9 rats for each) to generate a wound of 2 × 2 cm^2^. Subsequently, a hUCMSCs-loaded CCLDADM scaffold was spread on the wound, followed by daily injection of the drugs at the scaffold edge: 200 µL physiological saline for group A and B, 200 µL physiological saline containing 100 ng/mL Wnt3a for group C, and 200 µL physiological saline containing 100 ng/mL sFRP3 for group D. In 1, 2, and 3 weeks postoperatively, wound tissues were obtained to perform pathological examination of wound healing with the use of hematoxylin and eosin staining, and the wound area of each group was imaged and recorded via Image-Pro Plus 5.1 image analysis system. Inhalation anesthesia (2% isoflurane) was given during wound assessment and injection, and painkiller such as carprofen (2.2 mg/kg/day) was given after debridement procedures. The wound healing rate was calculated by the following equation.

$$ \begin{aligned} {\text{Wound healing rate}} & = \left( {{\text{the initial wound area}} -\, {\text{the residual wound area by day }}n} \right) \\ & \quad /{\text{the initial wound area}} \times 100\% . \\ \end{aligned} $$

#### Cell culture and groups

Detailed procedures of isolation and culture of hUCMSCs were reported in previous publications [9]. hUCMSCs were cultured in 10% FBS-containing complete media (normal culture medium supplemented with EGF, bFGF, insulin, retinoic acid, and CaCl_2_), replaced every 4 days. Based on drug intervention, the cells were allocated into three groups: control, Wnt activation (100 ng/mL Wnt3a added into culture media), and Wnt suppression (100 ng/mL sFRP3 added into culture media). Cell proliferation was assessed by BrdU assay on passages 2, 4, and 6.

#### Seeding of hUCMSCs on the CCLDADM scaffold

Detailed methods of CCLDADM scaffold preparing were reported in previous publications [6]. The CCLDADM scaffold was placed on a 6-well plate and sterilized with cobalt 60, followed by overnight incubation with the basal medium. On the following day, liquid on the scaffold surface was gently absorbed with sterile filter paper, and 50 µL of the hUCMSCs perfusion solution was added to the scaffold. Then, the 6-well plate was incubated for 1 h with 5% CO_2_. Fresh complete media were added into the plate when the cells attached to the scaffold, followed by replacing the media in 24 h and treatment with different drugs.

#### Cell proliferation assay with BrdU staining

The ratio of BrdU-positive cells was measured as follows: 1.5 × 10^5^/mL cells were seeded on a coverslip, and 30 μM BrdU was added at the log phase proliferation, followed by 6-h incubation in the dark. Subsequently, the cells were immobilized for 30 min post-fixation, permeabilized, and subjected to DNase treatment for 30 min at 37 °C. Finally, the cells were stained with BrdU antibody for 30–60 min in the dark at 4 °C, and the nucleus stained with DAPI (0.5 μg/mL). Images were captured under a fluorescence microscope, and total cells and BrdU-positive cells enumerated in three random vision fields for the calculation of positive cell ratio.

#### CCK-8 cell proliferation assay

1 × 10^5^ hUCMSCs cells were seeded on the 96-well plate and incubated with 10 µL CCK-8 reagent for 4 h before treatment with Wnt agonist or antagonist. The absorbance was measured at 450 nm. The faster the cell proliferation, the darker the color and the greater the absorbance values.

#### Live/dead staining

The staining procedures were as follows: 1 μL solution A (1 mM Live-Dye) and 1 μL solution B (1 mg/mL PI) were mixed in 1 mL staining buffer. hUCMSCs were rinsed three times with PBS prior to incubation with the prepared live/dead staining solution for 15 min at 37 °C and observed under laser confocal fluorescence microscopy. Green color indicated surviving cells while red indicated dead cells.

#### TUNEL assay

VasoTACS kit (R&D Systems, Minneapolis, USA) was employed to measure cell apoptosis in the tissues according to the kit manual. Briefly, terminal deoxynucleotidyl transferase was used to add biotinylated nucleotides to the DNA breakage with free 3′-hydroxyl residues, followed by subsequent detection with biotin-coupled BrdU antibody or Streptavidin-HRP. The nucleus was counterstained with DAPI, and images captured using a laser confocal microscope [10].

#### Evaluation on hUCMSCs differentiation

The assessment of hUCMSCs differentiation degree was based on their morphology. The epidermal stem-like cells are typically round or small polygons, and the ratio of cells with these morphologies was calculated to deduce the ratio of hUCMSCs differentiating into epidermal stem cells.

#### Western blot

Tissue or cells were homogenized in RIPA buffer (150 mmol/L NaCl, 5 mmol/L EGTA, 0.1% sodium deoxycholate, 1 mmol/L phenylmethylsulfonyl fluoride, 50 mmol/L Tris–HCl, and 0.1% SDS, pH 7.2) and stand on ice for 30 min. Lysates were centrifuged at 12,000×*g* for 10 min at 4 °C and the supernatant was collected. The protein concentration of the supernatants was determined by bicinchoninic acid assay. Equal amount of protein (20 μg) was separated by 10% SDS-PAGE and transferred to polyvinylidene difluoride (PVDF) membrane. Membranes were blocked with 3% BSA in TBST for 1 h and incubated with primary antibodies overnight at 4 °C, then washed with TBST and developed with an alkaline phosphatase color development kit. Bands were visualized with an enhanced chemiluminescence kit (Millipore, Billerica, MA, USA). Images were captured by ChemiDoc™ (Bio-Rad) with Image Lab software. The densities of blots were quantified using Quantity One analysis software [11].

#### Statistical analysis

All data were analyzed using SPSS13.0. Two samples were compared using Student’s *t* test, while that of multiple samples was performed using LSD (least significance difference) test and one-way ANOVA. Measurement data were expressed as mean ± standard deviation ($\bar{x}$ ± SD), and *P* < 0.05 was considered statistically significant.

## Results

### The growth characteristics of hUCMSCs seeded on the CCLDADM scaffold

**Fig. 1:**
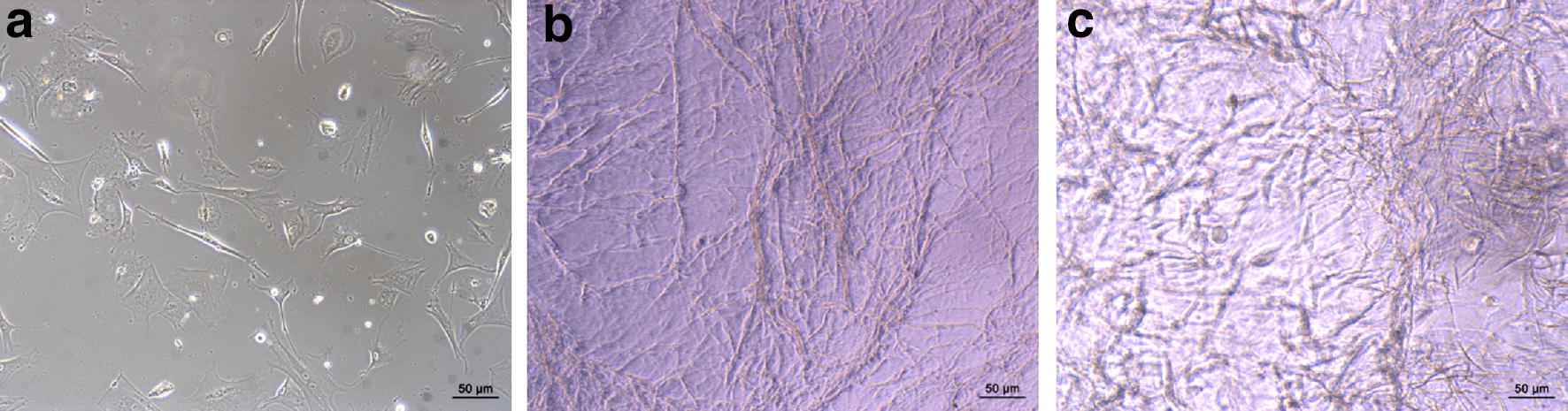
hUCMSCs morphology and growth characteristics on the CCLDADM scaffold. **a** hUCMSCs morphology under optical microscope (scale: 50 μm). **b** CCLDADM scaffold shape (scale: 50 μm). **c** Morphology of hUCMSCs after seeding on the CCLDADM scaffold (scale: 50 μm)

hUCMSCs were purified, and rod-like or irregular cord cell morphology was observed under normal culture. Along with an increasing number of passages, hUCMSCs presented the morphology of fibroblasts (Fig. 1a). The CCLDADM scaffold was shown to be uniform grids, with about 100 μm pore size (Fig. 1b). hUCMSCs presented polygon or irregular morphology with uniform growth, after seeding on the CCLDADM scaffold (Fig. 1c).

### Activation of the Wnt signaling pathway improved the therapeutic effect of hUCMSCs-loaded CCLDADM scaffold on diabetic wounds

**Fig. 2:**
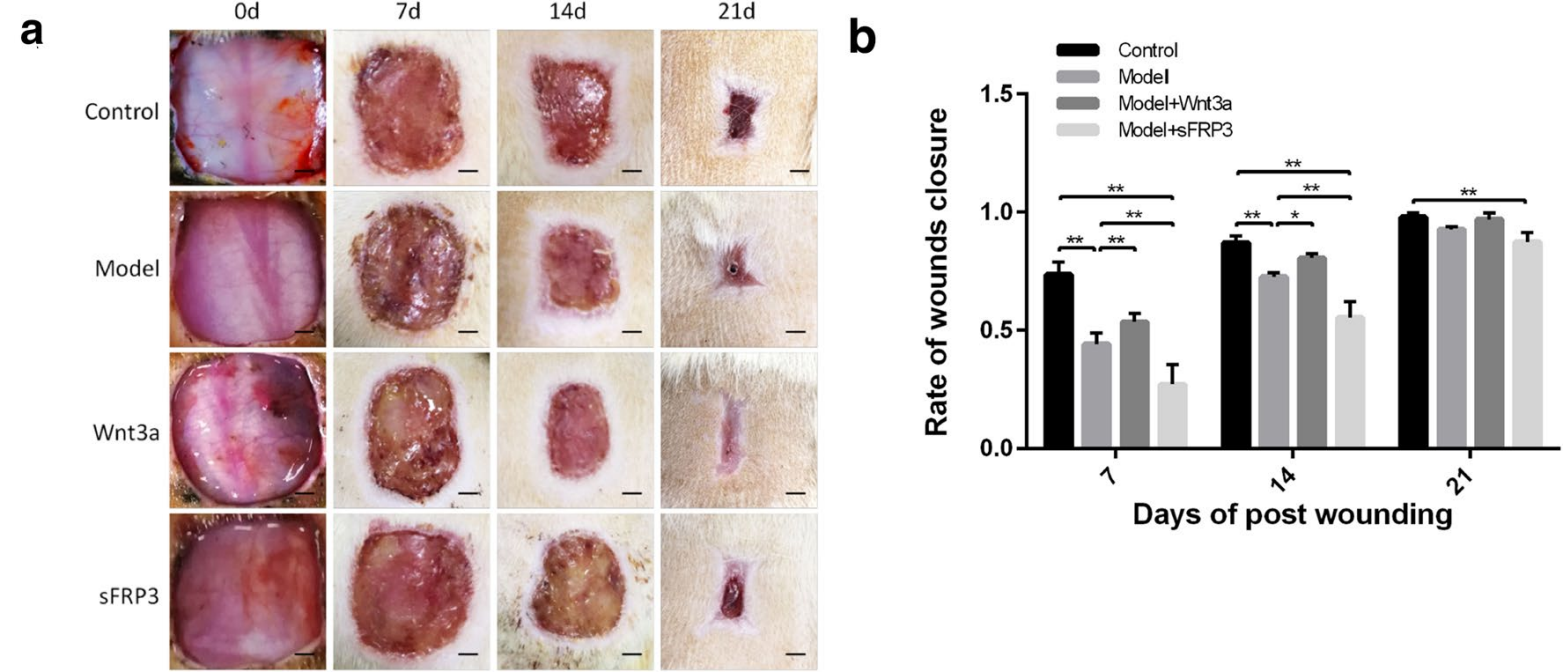
Activation of the Wnt signaling pathway improved the therapeutic effect of hUCMSCs-loaded scaffold for diabetic wounds. **a** Wound healing in different intervention groups on days 7, 14, and 28 postoperative (scale: 0.25 cm). **b** Statistical analysis of results in **c** (*n* = 6); *P < 0.05, **P < 0.01

The skin wound healing area was measured on days 7, 14, and 21 postoperative and the rate of wound healing was found to be significantly lower in diabetic rats than that in normal rats on days 7 and 14. This phenomenon was significantly improved by the activation of the Wnt signaling pathway in diabetic rats, while it was slowed by the blockade of the pathway (Fig. 2a, b).

### Activation of the Wnt signaling pathway promoted the generation of granulation tissues

**Fig. 3:**
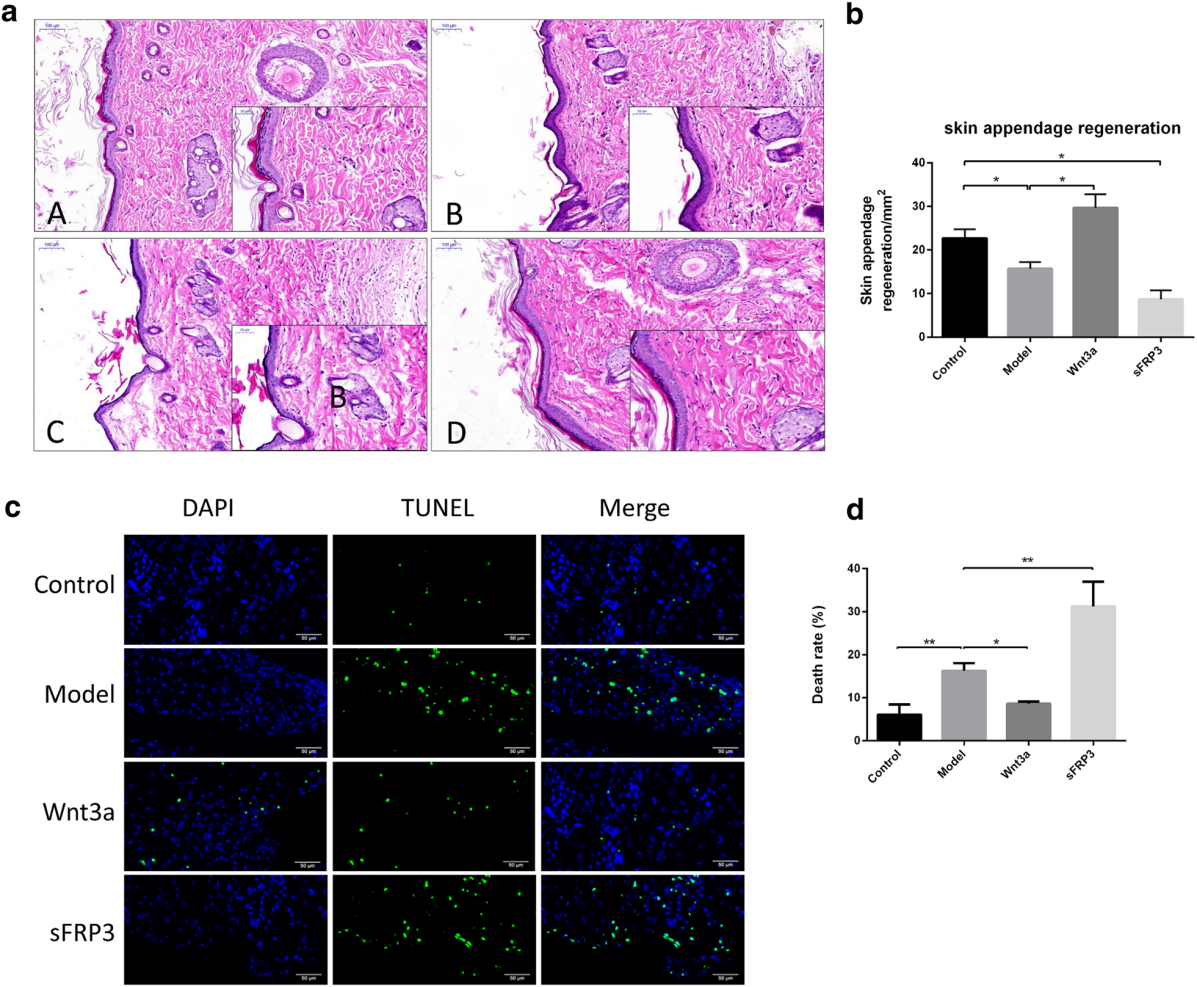
Activation of the Wnt signaling pathway promoted the generation of granulation tissues. **a** HE staining demonstrated the collagen fiber deposition and changes in capillaries in the granulation tissues (scale: 100 μm). **b** Statistical analysis of **a** results. **c** TUNEL staining detected cell apoptosis at the wound (scale: 50 μm). **d** Statistical analysis of **c** results (*n* = 3); *P < 0.05, **P < 0.01

Hematoxylin–eosin (HE) staining examined the growth of the granulation tissue at the wound. The results revealed that new blood vessels and integumentary appendages at the wound were less in diabetic rats than the normal rats, while the activation of the Wnt signaling pathway improved (sFRP significantly suppressed) the thickness of the granulation tissues, the deposition of fiber tissues, and the number of capillaries (Fig. 3a, b). Moreover, the TUNEL assay displayed that cell apoptosis increased in the diabetic rats at the wound granulation tissues that was significantly decreased by the activation of the Wnt signaling pathway (sFRP further enhanced the decrease) (Fig. 3c, d).

### Activation of the Wnt signaling pathway promoted hUCMSCs survival on the CCLDADM scaffold

**Fig. 4:**
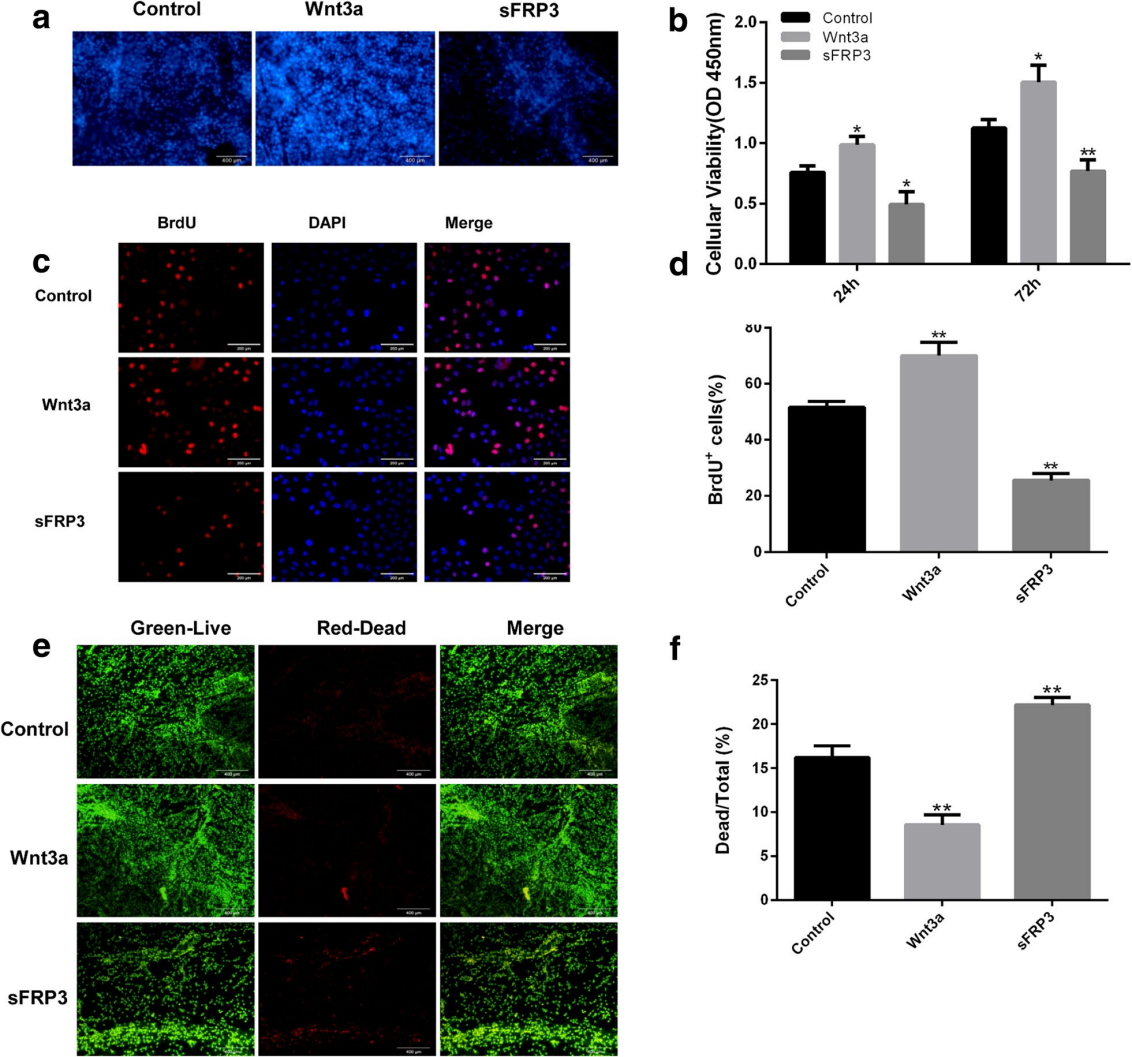
Activation of the Wnt signaling pathway promoted hUCMSCs survival on the CCLDADM scaffold. **a** hUCMSCs were seeded on the CCLDADM scaffold and treated with Wnt3a or sFRP3 for 72 h, followed by DAPI staining to estimate the total number of cells on the scaffold (scale: 200 μm). **b** CCK-8 staining detected the total number of cells on the CCLDADM scaffold at 24 or 72 h post-drug intervention (*n* = 3). **c** hUCMSCs were seeded in a 6-well plate and treated for 72 h with Wnt3a or sFRP3, and the ratio of BrdU-positive cells was detected to reflect the rate of proliferation (scale: 100 μm). **d** Statistical analysis of BrdU-positive ratio from **c** (*n* = 3). **e** Live/dead cell staining evaluated the ratio of dead cells on the CCLDADM scaffold (scale: 100 μm). **f** Statistical analysis of results from **e** (*n* = 3); *P < 0.05, **P < 0.01 vs. control

hUCMSCs were seeded on the CCLDADM scaffold, cultured in vitro, and stimulated with Wnt3a and sFRP for 72 h. DAPI staining demonstrated a significant cell number in the Wnt3a group as compared to the control group, whereas less cell number in the sFRP group than the control (Fig. 4a, b). CCK-8 staining was utilized to label the surviving cells, which displayed that hUCMSCs proliferated significantly faster on the CCLDADM scaffold in the Wnt agonist group than the control group, whereas intervention with Wnt antagonist resulted in a significant decrease (Fig. 4c, d). To further validate these results, BrdU staining was employed to measure the proliferation of hUCMSCs during in vitro culture; the result was in agreement with previous data. Live/dead cell staining revealed that the activation of Wnt signaling pathway significantly decreased the ratio of dead cells, whereas the blockade of the pathway further aggravated the phenomenon (group control: Wnt3a: sFRP3 = 16.23 ± 1.31%:8.57 ± 1.13%:22.19 ± 0.83%) (Fig. 4e, f).

### Activation of the Wnt signaling pathway enhanced hUCMSCs differentiation

**Fig. 5:**
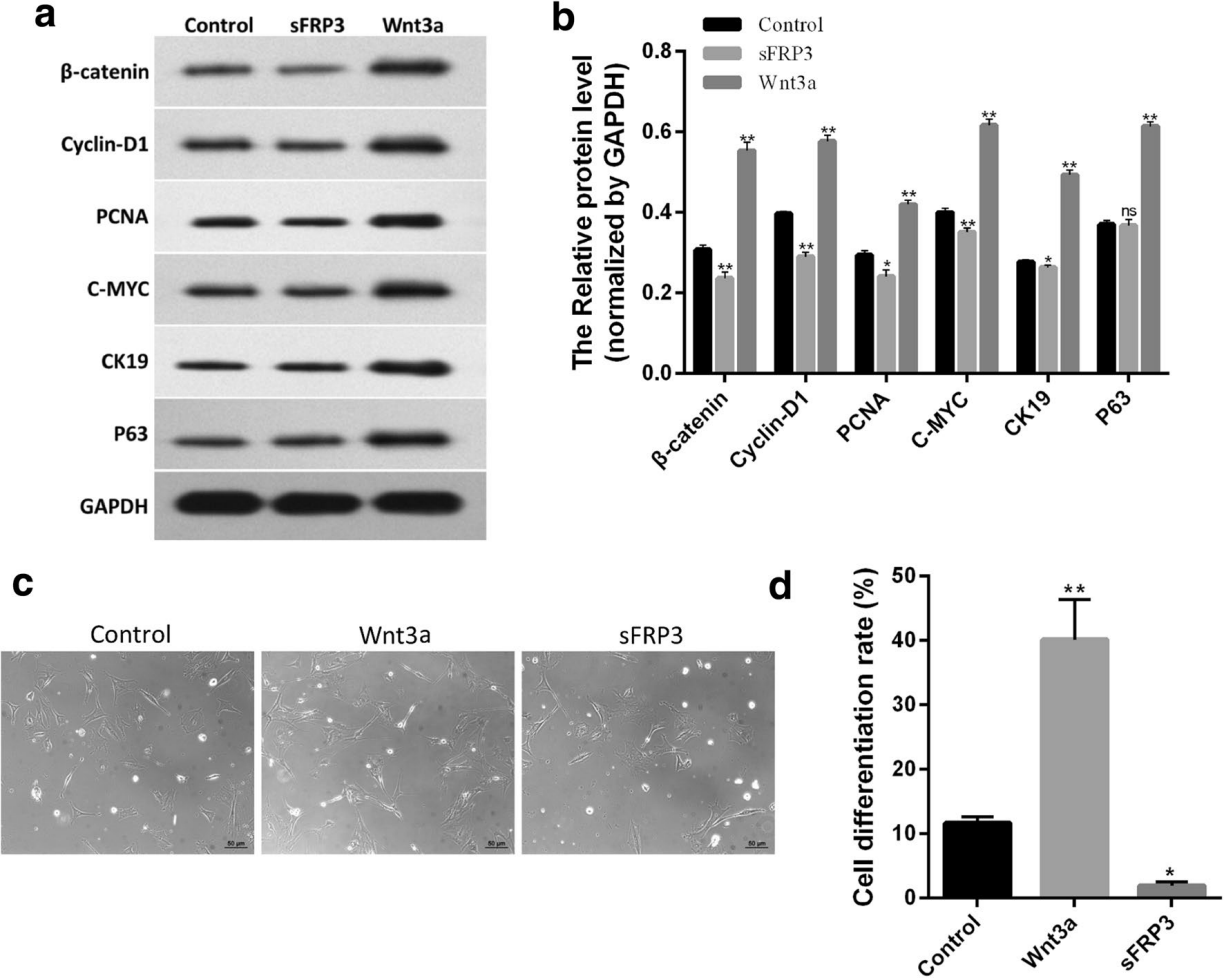
Activation of the Wnt signaling pathway enhanced hUCMSCs differentiation. **a** Western blotting was applied to detect the changes in the levels of β-catenin, c-Myc, p63, CK19, and PCNA proteins. **b** Statistical analysis of **a** results. **c** The differentiation degree of hUCMSCs was evaluated by cell morphology under optical microscope (scale: 100 μm). **d** Statistical analysis on the ratio of cells with round or small polygonal morphology displayed in **c**; *P < 0.05, **P < 0.01 vs. control (*n* = 3)

The differentiation of hUCMSCs towards epidermal cells is critical for the regulation of tissue repair [12]. To explore whether Wnt signaling pathway was involved in hUCMSCs differentiation, these cells were treated for 72 h with Wnt3a or sFRP. Western blotting data displayed that Wnt3a significantly increased the levels of β-catenin, c-Myc, p63, CK19, and PCNA proteins, whereas sFRP significantly suppressed the expression of these proteins, indicating that Wnt3a and sFRP activated and suppressed Wnt signaling pathway, respectively (Fig. 5a, b). Morphology analysis demonstrated that the ratio of cells with round or small polygonal morphology increased after activation of Wnt signaling pathway, indicating a rise in the differentiation of hUCMSCs towards epidermal stem cells; conversely, sFRP treatment led to a significant reduction in the ratio of these cells (Fig. 5c, d). These results implied that the activation or suppression of Wnt signaling pathway played a regulatory role in hUCMSCs differentiation.

## Discussion

Diabetes mellitus is the leading chronic disease posing risks to human health. It not only compromises the quality of patient’s life but also gives rise to an economic burden on society and families [2]. The primary manifestation of diabetic foot is chronic skin ulcers on lower limbs, and a subset of patients with chronic ulcers require amputation; refractory diabetic ulcers constitutes 50–70% of all amputations [13]. Hitherto, the mechanism underlying the incurable skin wound in diabetic patients remains unclear; however, some theories propose the involvement of neuropathy, vascular remodeling, inflammatory reaction, and metabolic abnormality of the local wound [14]. In addition to the conventional preventive measures, stem cell therapy is gradually emerging in the management of chronic diabetic ulcers [15–17]. Previous studies displayed that MSCs promoted the healing of mouse cutaneous wound [18, 19]. Nonetheless, MSCs have rarely been reported to be successful on humans. This failure might be attributed to the following: (1) intravenous perfusion of MSCs pose great safety risks; (2) when MSCs are injected subcutaneously around the wound area, differentiated MSCs cannot migrate easily to the wound; (3) only a small portion of MSCs cells smeared on the wound can attach and survive [20, 21]. In the previous study, we adopted CCLDADM as a scaffold on skin wound to enhance stable proliferation of hUCMSCs and capillary regeneration at the wound site [6]. However, this method on diabetic ulcers still faces considerable challenges due to incurable diabetic wounds.

Furthermore, Wnt signaling pathway is highly conserved across evolution, suggesting its essential role in embryonic development and stem cell growth and differentiation [22]. β-Catenin is the canonical effector molecule in the Wnt signaling pathway [23]. Wnt protein, when bound to its membrane receptors, such as FZD and LRP5/6, elicits the dissociation of β-catenin from the degradation complex, thereby attenuating its phosphorylation catalyzed by GSK3β and CK1 and diminishing the subsequent ubiquitination. Also, the activation of Wnt signaling pathway enhances the nuclear translocation of β-catenin, which then regulates the expression of target genes, such as *cyclin*-*D1*, *c*-*Myc*, *p63*, *CK19*, and *PCNA*, which regulate the cell proliferation and differentiation [23]. The binding of Wnt protein to its receptors is modulated by a variety of molecules: sFRPs and Wnt inhibitory factor (WIF-1). These two proteins can competitively inhibit the binding of Wnt3a to FZD via interaction with FZD, thereby restraining the stimulation of Wnt signal [22]. The abnormal Wnt signal is closely associated with multiple diseases, such as malignancies, liver fibrosis, rheumatoid arthritis, bone development, and immune response [24, 25]. The role of Wnt signaling pathway in above diseases is closely related to the regulation of cell proliferation and differentiation [25]. Previous studies reported the attenuation of Wnt signal in diabetic nephropathy and diabetic ulcer wounds, implying its role in the pathogenesis of the two diseases [8, 26]. To further explore whether Wnt pathway could modulate the therapeutic effect of hUCMSCs-loading matrix scaffold material for diabetic ulcers, the present study applied hUCMSCs-seeding CCLDADM scaffold on the skin ulcer wounds of both normal and diabetic rats. Moreover, Wnt signal agonist or antagonist was administered at the scaffold edge. The results showed that the healing rate of skin wound was significantly lower in diabetic rats than that in the normal control, along with a reduction in protein levels of cyclin-D1, c-Myc, p63, CK19, and PCNA, indicating attenuated Wnt signaling pathway [27]. In addition, we found that the healing of skin wound was significantly accelerated in diabetic rats, following the activation of the Wnt signaling pathway, while it significantly slowed down post-suppression of the signal. The results of HE staining demonstrated that the activated pathway raised the abundance of new blood vessels and integumentary appendages in the granulation tissue, further proving that the activation of Wnt signaling pathway could enhance the therapeutic effect of the CCLDADM scaffold on diabetic ulcers.

In the present study, Wnt3a and sFRP were injected at the scaffold edge, rendering the possibility that Wnt3a and sFRP might exert the biological effect through rat innate somatic cells at the wound edge. To validate that Wnt3a and sFRP could regulate the hUCMSCs proliferation and differentiation seeding on the CCLDADM scaffold, the hUCMSCs-seeding scaffold was cultured in vitro and stimulated with Wnt3a and sFRP. The results showed that Wnt3a could promote and sFRP could suppress the proliferation and differentiation of hUCMSCs, and Wnt3a decreased the hUCMSCs death, thereby increasing the survival number of hUCMSCs on the CCLDADM scaffold.

Thus, hUCMSCs combined with CCLDADM scaffold for the treatment of refractory diabetic ulcers exhibit several advantages, such as potent differentiation of hUCMSCs and abundant source of umbilical cord without ethical issues. Furthermore, increasing development in material science proposes multiple options for engineered skin materials. The key study orientations for the next step focus on the selection of the scaffold material, improvement of the proliferation and differentiation efficiency of hUCMSCs seeded on the engineered skin, and the safety of the usage of hUCMSCs in humans.

## Conclusions

In summary, this study implied that changes in the Wnt signaling are critical for the healing of the diabetic wound, and activation of the pathway can improve the therapeutic effect of CCLDADM scaffold on the diabetic wound. Therefore, this study provides new insights into the improvement of the therapeutic effect of skin tissue engineering using hUCMSCs for chronic diabetic ulcers.
